# Parent-Mediated Interventions in Infants at Risk of Autism Specturm Disorder: A Neuropsychiatric Systematic Review and Meta-Analysis

**DOI:** 10.1192/j.eurpsy.2026.11138

**Published:** 2026-07-31

**Authors:** L. Kade, H. B. Needham

**Affiliations:** 1 Milton Keynes University Hospital, Milton Keynes; 2 Norwich Medical School, Norwich, United Kingdom

## Abstract

**Introduction:**

Autism Spectrum Disorder (ASD) is a neurodevelopmental condition characterised by social communication deficits. The diagnosis, treatment and ASD research focuses on children >24 months, though risk is detectable in infancy. Parent-mediated interventions (PMIs) promote early social and cognitive development, but the impact during infancy remains unexplored. This systematic review and meta-analysis found PMIs delivered to high-risk infants (<15 months) can improve early social communication and modestly reduce symptom severity, potentially influencing developmental trajectories.

**Objectives:**

Analyse PMI effectiveness in high-risk infants ≤15 months.

Examine PMI as an early neuropsychiatric intervention, exploring the neurobiological mechanisms behind its effects and limitations.

Evaluate the feasibility of PMI within local healthcare settings, and pre-existing 12-15 month national paediatric health visitor pathways.

**Methods:**

The review followed PRISMA guidelines. Databases searched included Medline, COCHRANE, Scopus, APA PsycINFO, and PubMed (last search February 2025). Risk of bias was assessed using RoB 2.0 and the Newcastle-Ottawa Scale. Studies evaluating PMI efficacy in high-risk infants ≤15 months were included, exclusion criteria were participants >15 months or no consideration of PMI efficacy. A random-effects meta-analysis was conducted.

**Results:**

Of 259 studies screened, nine randomised-controlled trials and longitudinal studies (n = 247 infants) met inclusion criteria. PMIs produced moderate-to-large improvements in social communication and parent–child engagement, pooled effect size (Hedges’ g = 0.58, 95% CI 0.53–0.63, p = 0.001). Significant improvements occurred in social engagement (g = 0.44, p = 0.002) and joint attention (g = 0.66, p = 0.001). PMI modestly reduced ASD symptom severity (g = –0.12, p = 0.011), with effects peaking at 16–20 weeks of intervention. Therapist-supported models (iBASIS-VIPP) showed the greatest impact (SMD = 0.51), followed by hybrid (0.42) and self-directed (0.32) formats; adherence was highest with therapist-led delivery (78%). Remote models demonstrated sustained but smaller benefits. Overall, findings highlight promising, early improvements in social communication and engagement, an area where meaningful shifts in diagnostic outcomes remain rare.

**Conclusions:**

Despite small samples, lack of RCTs, and limited long-term data, this review suggests PMIs are a promising neuropsychiatric intervention for high-risk ASD infants (≤15 months). Evidence indicates PMIs can improve early social development, communication, and quality of life while reducing later psychiatric risk and ASD symptom severity. PMIs mark a shift in autism care; early intervention uses proactive and personalised neuropsychiatric care rooted in neuroscience. Future studies should assess long-term outcomes, optimal duration, cost-effectiveness, and feasibility.

**Disclosure of Interest:**

None Declared

